# Characteristics of PEO Incorporated with CaTiO_3_ Nanoparticles: Structural and Optical Properties

**DOI:** 10.3390/polym13203484

**Published:** 2021-10-11

**Authors:** Shujahadeen B. Aziz, Muaffaq M. Nofal, Mohamad A. Brza, Sarkawt A. Hussein, Khaled H. Mahmoud, Zeinhom M. El-Bahy, Elham M. A. Dannoun, Wrya O. Kareem, Ahang M. Hussein

**Affiliations:** 1 Hameed Majid Advanced Polymeric Materials Research Lab, Physics Department, College of Science, University of Sulaimani, Qlyasan Street, Kurdistan Regional Government, Sulaimani 46001, Iraq; mohamad.brza@gmail.com (M.A.B.); sarkawt.hussen@univsul.edu.iq (S.A.H.); ahang.hussein@univsul.edu.iq (A.M.H.); 2 Department of Civil Engineering, College of Engineering, Komar University of Science and Technology, Kurdistan Regional Government, Sulaimani 46001, Iraq; 3 Department of Mathematics and General Sciences, Prince Sultan University, P.O. Box 66833, Riyadh 11586, Saudi Arabia; muaffaqnofal69@gmail.com; 4 Department of Physics, College of Khurma University College, Taif University, P.O. Box 11099, Taif 21944, Saudi Arabia; k.hussein@tu.edu.sa; 5 Department of Chemistry, Faculty of Science, Al-Azhar University, Nasr City, Cairo 11884, Egypt; zeinelbahy2020@yahoo.com; 6 Associate Director of General Science Department, Woman Campus, Prince Sultan University, P.O. Box 66833, Riyadh 11586, Saudi Arabia; elhamdannoun1977@gmail.com; 7 Department of Chemistry, College of Science, University of Sulaimani, Qlyasan Street, Kurdistan Regional Government, Sulaimani 46001, Iraq; wrya.karim@univsul.edu.iq

**Keywords:** PEO, polymer nanocomposites, XRD test, optical properties, refractive index, band gap study

## Abstract

In this research, direct band gap polymer composites with amorphous phase, which are imperative for optoelectronic devices applications were synthesized. The solution cast technique was used to produce polyethylene oxide (PEO)/calcium titanate (CaTiO_3_) nanocomposite (NC) films. The X-ray diffraction (XRD) confirms the growth of amorphous nature within PEO with CaTiO_3_ addition. The optical band gaps of pure PEO and PEO/CaTiO_3_ NC films were calculated using analysis of ultraviolet–visible (UV-Vis) spectra. The change in absorption edge toward lower photon energy is evidence of polymer modification. The dispersion behavior of the refractive index of PEO was manipulated to a higher wavelength upon doping with CaTiO_3_. Upon adding CaTiO_3_ to the pure PEO polymer, the dielectric constant and refractive index were considerably modified. The band gap shifts from 4.90 eV to 4.19 eV for the PEO incorporated with an optimum portion of 8 wt. % of CaTiO_3_. The types of the electronic transition in composite samples were specified, based on the Taucs model and the optical dielectric loss. The alteration of UV/Vis absorption spectra of the NC film was considered a suitable candidate to be applied in nanotechnology-based devices. The spherulites ascribed to the crystalline phase were distinguished through the optical microscopy (OM) study.

## 1. Introduction

Investigation of remarkable mechanical, electrical, and optical properties of polymer composites in specific applications, such as flexible electronics or photonics, is a highly active area of research [1]. As a principle, optical technologies, for example, light-emitting and solar cell devices, strongly rely on the interaction between advanced materials and light. It is also documented that polymer materials can be utilized in light-emitting diodes (LEDs), optical devices, and sensors. This is due to their attractive optical properties. Manipulation of polymer optical characteristics can be achieved simply by optimizing the appropriate dopant materials and concentrations. Several optical properties are mentioned herein, such as extinction coefficient, energy gaps, optical loss, and dielectric constants. These can be determined based on the significant optical parameters of polymeric film absorbance, transmittance, and reflectance.

Based on the literature, the calculation of the absorption coefficient, refractive index, extinction coefficient, and real and imaginary parts of the dielectric constant can easily be performed [2]. Polymer-based materials have desired optical and electrical properties. These materials have taken positions as materials of choice for various applications, such as batteries, solar cells, fuel cells, and capacitors [3,4,5,6]. Modifications in polymer materials involve electrical and optical properties, which, in turn, allows these materials to perform multifunctions. In principle, alteration in structure causes modification to the optical properties of polymers by fabricating nano-size materials. The dopant addition that causes the mechanism of electron transitions to change and reduces the energy band gap, is included in this structural modification [7]. From the analysis of optical properties, one can gain insight into the transition of electrons between conduction and valence bands in polymers. Furthermore, a comprehensive understanding of the charge transport phenomenon in polymers is gained from the electrical conduction property [8,9]. Based on previous works, polymers’ optical and electrical properties can be enhanced by incorporating metals and semiconductor particles [10,11,12,13]. The addition of fillers influence host materials are diverse; therefore, it is crucial to add proper fillers with optimum quantity [14]. The PEO consists of a linear structure that possesses semicrystalline polymer that contains a crystalline phase and amorphous phase at ambient temperature; however, it has a crystalline structure in its pure form [15]. PEO-based materials are promising polymer candidates because of their relatively high thermal stability [15]. It has several desired characteristics, such as satisfactory dimensional stability, good conductivity in the amorphous structure, cost-effectiveness, and sufficient corrosion resistance [3]. Moreover, it has a strong tendency to form a complex. All these factors make PEO an appropriate polymer electrolyte [16]. Furthermore, the PEO can be used as a host material for solid polymer electrolytes without solvents [1]. However, the ionic conductivity of PEO is not as high as required because of the abundance of crystalline phase in the structure, which is a challenging factor to reach the desired DC conductivity [2,15]. In general, the superiority of polymers comes from the transparency, cost-effectiveness, light weight, ease of processing, and robust mechanical property. Nevertheless, the PEO is the most suitable candidate to be applied in the optics area. One of the drawbacks of utilization this material is a relatively low index of refractive [7,17].

To improve the property of polymeric materials, the CaTiO_3_ was used. CaTiO_3_ is mostly applied in specific fields of electronic ceramics containing specific electronic ceramics, a positive temperature coefficient, and a ferroelectric ceramic capacitor [18,19]. CaTiO_3_, a vital lead-free Perovskite material, has newly attracted more attention by researchers due its interesting properties and different industrial applications [20]. Muhammed et al. [21] studied the structure and optical properties of PEO doped with tin titanate (SnTiO_3_) nano-filler using XRD and UV-Vis spectroscopy. The authors determined that the crystalline phase of PEO was decreased using SnTiO_3_. They also showed that the optical parameters, such as dielectric constant, refractive index, and absorption coefficient increased, while the optical energy band gap decreased. Hussen et al. [22] prepared nanocomposite polymer of polystyrene (PS) doped with SnZrO_3_ nanoparticle. The author used XRD and UV–Vis spectroscopy to investigate crystallinity and optical properties of the pure PS and nanocomposite films. The author showed that the amorphous phase and optical property were enhanced.

The aim of this study was directed toward creating new band gap energy using PEO incorporated with CaTiO_3_ nanoparticles. Tremendous work was required to achieve the hypothesis of introducing band gap energy levels. This study used the dielectric loss plot for determining the optical energy bandgap and Tauc’s model for identifying the types of electron transition. Several theoretical principles and experimental approaches were applied to prove the effectiveness of optical dielectric loss in determining the electron transition nature from the Tauc’s model. Hence, from the investigation of the band gap, it was apparent that earlier studies could not answer the questions around this topic. Additionally, these findings can serve as the basis for developing new approaches for manipulating optical properties via band gap alteration within the area of polymers and condensed matters.

## 2. Experiments

### 2.1. Polymer Composite Preparation

**Figure 1:**
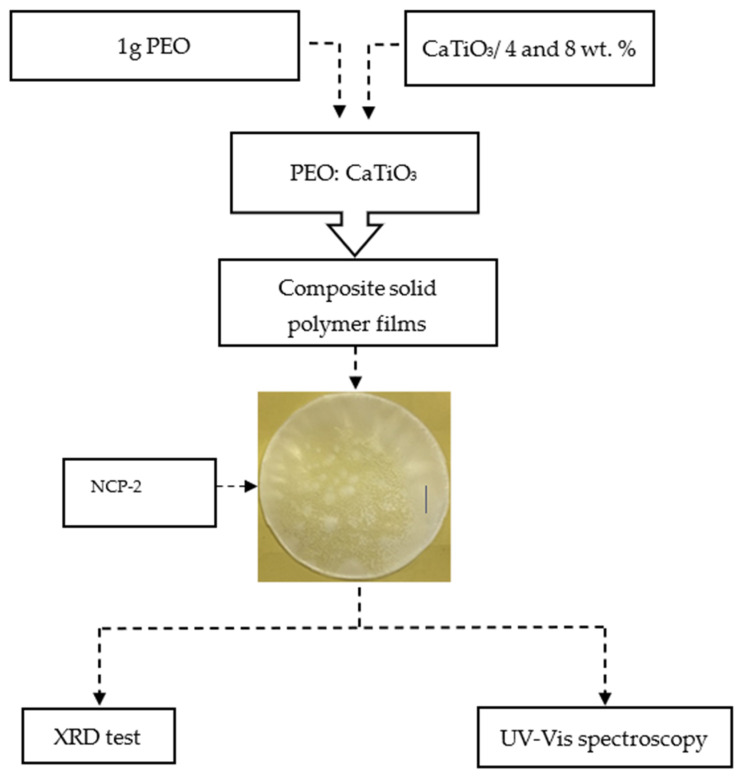
Flowchart of the composite preparation process and picture of composite membrane.

Polyethylene oxide (PEO) as a raw material in powder form was used in this work. It has a molecular weight > 5 × 10^6^ g·mol^−1^ and was bought from Sigma-Aldrich. The solution-casting technique was implemented in the film formation. The preparation of the solution of PEO polymer comprised the addition of 1 g of PEO powder into 50 mL distilled water. The mixture was stirred using a magnetic stirrer for five hrs at ambient temperature. When the solution of the polymer was gained as a clear viscous solution, two portions of 4 and 8 wt.% of CaTiO_3_ were added into two separate containers. Then, the stirring continued until the formation of the PEO/CaTiO_3_ polymer composite. The pure PEO and PEO-4 wt.% and 8 wt.% CaTiO_3_ were labelled as NCP-0, NCP-1, and NCP-2, respectively. Ultimately, plastic Petri dishes were used to cast the solutions by allowing them to dry at ambient temperature. Subsequently, the films were maintained in desiccators, enriched in silica gel for further drying. The thickness of the films used for characterization were in the range between 0.025 and 0.03 cm. A pictorial fabrication process and image of composite membranes for the fabricated composite films are shown in Figure 1.

### 2.2. X-ray Diffraction (XRD)

The X-ray diffractometer (Empyrean XRD-Panalytical) operated at40-kV and 45-mA current, was used to gain the XRD at ambient temperature. A beam of X-ray with λ = 1.5406 A° was passed through each sample with the glancing angle ranged 10° ≤ 2θ ≤ 80°, in step size of 0.05°.

### 2.3. UV-Vis Measurement

A Jasco V-570 UV-Vis-NIR spectrophotometer (Japan, Jasco SLM-468) mode was employed to acquire the ultraviolet-visible (UV-vis) absorption spectra of the solid polymer films based on PEO.

## 3. Results and Discussion

### 3.1. XRD Analysis

**Figure 2:**
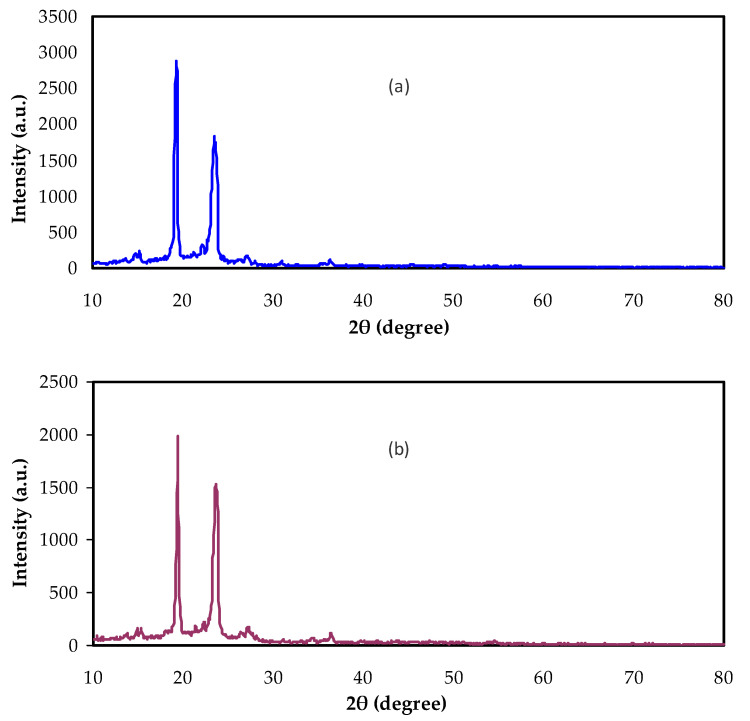

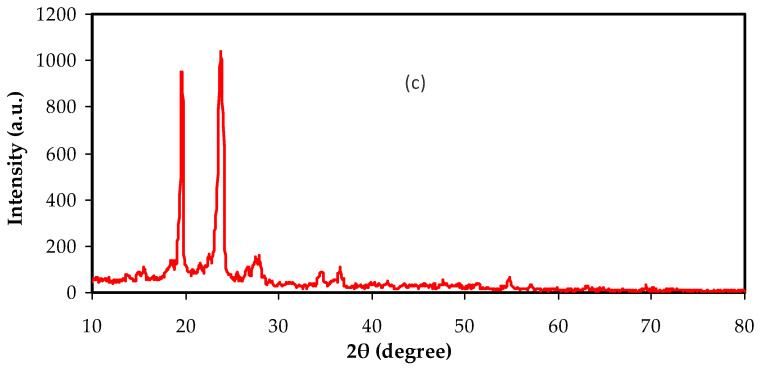
XRD spectra for (**a**) Pure PEO, (**b**) NCP-1, and (**c**) NCP-2.

Figure 2 shows the XRD spectra for the neat PEO and composite systems. In Figure 2a, the XRD pattern of pure PEO is shown. We noticed that the crystalline phase dominates that evidenced by the presence of two narrow peaks [23]. Two main domains were seen; firstly, two essential peaks at 18° and 24°, and low-intensity peaks at the high angle appear. PEO polymers’ crystalline and semi-crystalline structures can be recognized from these different diffraction peaks [24,25,26]. The structure of PEO is identified to be both semi-crystalline and linear. The stabilization of the PEO crystalline structure, electrochemically and chemically, was the structural unit’s responsibility. The existence of C-O, C-C, and C-H bonds in PEO caused the polymer to be stable chemically and mechanically [15]. Rajeh et. al. [26], stated that the peaks at around 22° and 18° referred to (112) and (120) planes.

The decrease and broadening of the intensity of the XRD peaks by the addition of the nano-size CaTiO_3_ are seen in Figure 2b,c. This confirms the increasing amorphous region at the expense of the crystalline structure in the PEO. Additionally, the complexation between the CaTiO_3_ and PEO can be emphasized. Furthermore, the dominancy of the amorphous phase facilitated polymer chain segmental motions [27].

The consequence of dopant addition was the decrease in the crystalline phase, i.e., lowering the compact nature of the polymer structure [28,29]. It was documented that the chemical stability of the PEO polymer crystalline structure is related to the building block units, such as C-H, C-O, and C-C bonds [28,30].

### 3.2. Absorption Study

**Figure 3:**
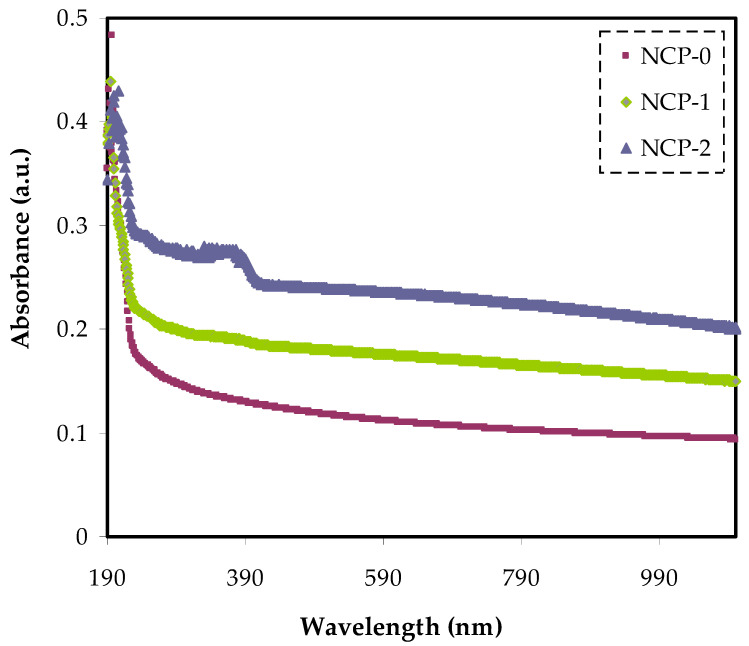
Absorption spectra of NCP-0 and composite samples.

The absorption spectra of pure PEO and doped PEO with various quantities of CaTiO_3_ nanoparticles in the wavelength, ranged between 180 and 900 nm, as shown in Figure 3. We noticed that the intensity of absorption in the spectrum of each sample decreased with increased wavelength, while it increased with increased CaTiO_3_ quantity. It is observed that, the films are almost transparent at the high wavelengths. In pure PEO, the spectrum contained an absorption edge at nearly 210 nm, and a noticeable shift in the absorption edge to a higher wavelength was recorded for each film. The absorption band for each sample under study in the wavelength, ranged between 200 and 300 nm, which could be ascribed to the occurrence of $\pi -\pi^{*}$ electronic transition, which was to be expected for conjugated (C = O) group [31,32]. Interestingly, the vibronic shoulder appeared clear as the quantity of CaTiO_3_ increased. Importantly, the similarity in shape of the PEO:CaTiO_3_ absorption spectra showed a desirable homogeneity of the films formed.

**Figure 4:**
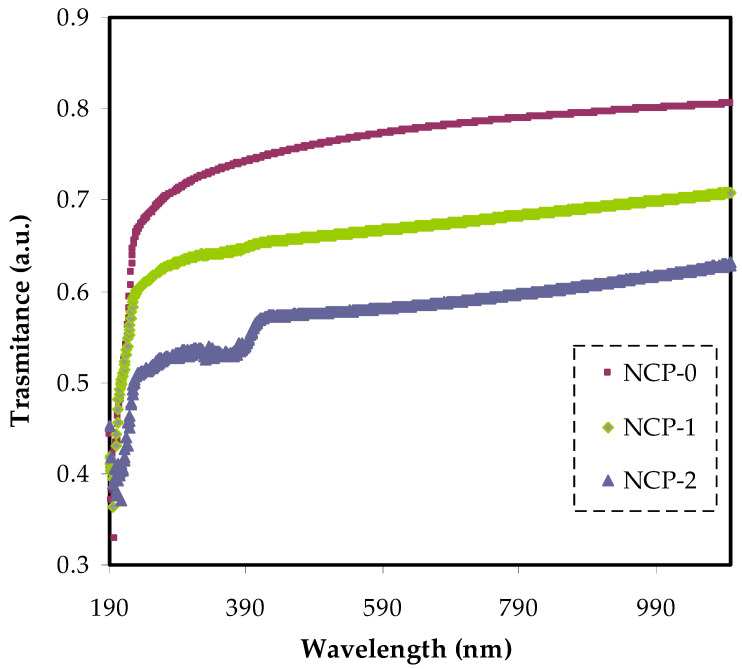
The transmittance of NCP-0 and composite samples.

Figure 4 also shows the transmittance of neat PEO and doped PEO with various quantities of CaTiO_3_ particles. The pure PEO showed relatively high transparency beyond the visible region exceeding 81%. On the other hand, below the visible region, the transparency dropped due to the high absorption of the films. It is worth noting that the doped PEO with 4 and 8 wt.% CaTiO_3_ showed lower transparency and reached almost 0.51% at the UV region and the transparency increased to 0.63% at the visible region. This can be explained based on the scattering and relatively high refractive index of highly doped films at lower λ (nm).

**Figure 5:**
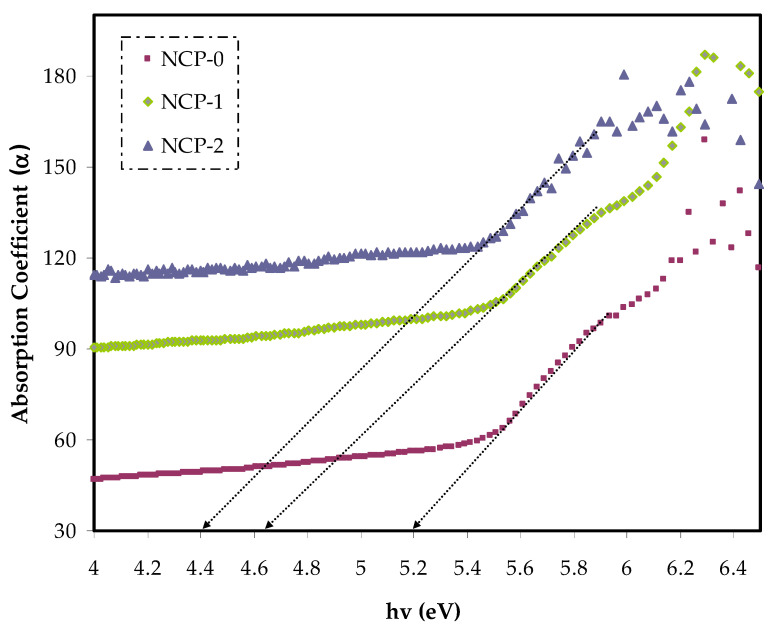
Absorption coefficient against photon energy for NCP-0 and composite films.

From the optical absorption spectrum, one can gain insight into the band gap energy and structure of non-crystalline and crystalline materials. One of the useful optical parameters was absorption coefficient (α), which can be described as quantifying the light amount that a medium can absorb. This was obtained from the incident radiation fraction absorbed per absorbent thickness [33]. The absorption coefficient of a series of prepared films of PEO and various doped PEO with CaTiO_3_ are shown in Figure 5. The straightforward way of calculating the absorption coefficient was carried out using the following equation [34]:

(1) $$\alpha =\frac{2.303A}{t}$$

where, *t* stands for the thickness of the sample and *A* stands for the absorbance. The cause of increasing (α) with increasing CaTiO_3_ content could be related to the modification of molecular configuration. In other words, it supported the process of charge transfer within metal ion complex systems, including the host polymer [35]. After extrapolating the linear part of the curves to zero absorption, we were able extract the absorption-edge value as tabulated in Table 1. In the current calculation, the absorption edge energy of pure PEO was lowered from 5.2 to 4.4 eV upon adding 8 wt.% of CaTiO_3_. This absorption edge lowering can be interpreted as enhancing interchain interaction between the polymer composite chains, upon increasing the dopant quantity. As a consequence, a denser conjugation stacking was produced. In other words, the formation of new effective trap levels in the optical band gap could be formed by changing the absorption edge. Therefore, electrons passed the top of the valence band to the bottom of the conduction band within these new states [36,37]. The current optical measurements were similar to those gained for PEO by Kumar et al. [38].

### 3.3. Refractive Index Study

In designing new materials, both a refractive index and optical dielectric constant were considered decisive parameters. For calculating the refractive index (*n*), Fresnel formulae was implemented for parent PEO film and PEO films doped with CaTiO_3_ using measured values of extinction coefficient (*k* = αλ/4π) and reflectance (*R*) [39]. It was imperative that the designing of optoelectronic devices relied significantly upon the accuracy of info on the refractive index parameter. In principle, the refractive index was related to both the mean polarizability and density of the medium at specific temperature and pressure [40].

Thus, the refractive index was a decisive parameter that determined the optical performance. The refractive index depended on absorption and reflectance. The complex refractive index of films was calculated by Equation (2):

*n* × (*λ*) = *n*(*λ*) + *k* (*λ*) (2)

where, the extinction coefficient is symbolized by *k* and the refractive index is referred by *n*. The following relationship shows the correlation between the *k* and *n* [41]:

(3) $$n=\left[\frac{\left(1+R\right)}{\left(1-R\right)}\right]+\sqrt{\frac{4\times R}{{\left(1-R\right)}^{2}}}-K^{2}$$

**Figure 6:**
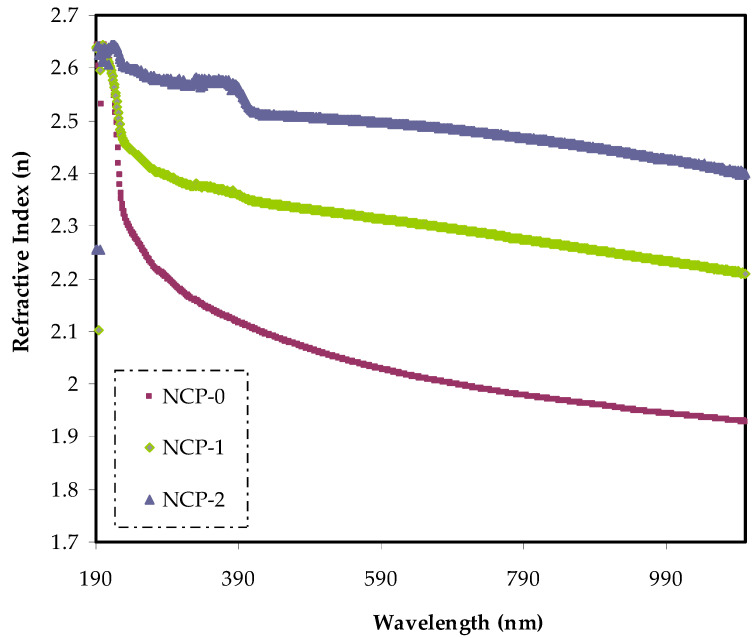
Refractive index v wavelength for NCP-0 and composite films.

The dispersion curves of the refractive index *n* (*λ*) of the PEO and PEO loaded with CaTiO_3_ are exhibited in Figure 6. In the spectrum of the parent PEO film, the dispersion region lay at a wavelength of less than 300 nm. In contrast, in the case of doped PEO films with CaTiO_3_, the refractive index displayed a shift in dispersion to a higher wavelength region. This was mainly related to the uniform CaTiO_3_ distribution throughout the PEO matrix, resulting in a greater density of the nanocomposite.

### 3.4. Band Gap Study

**Figure 7:**
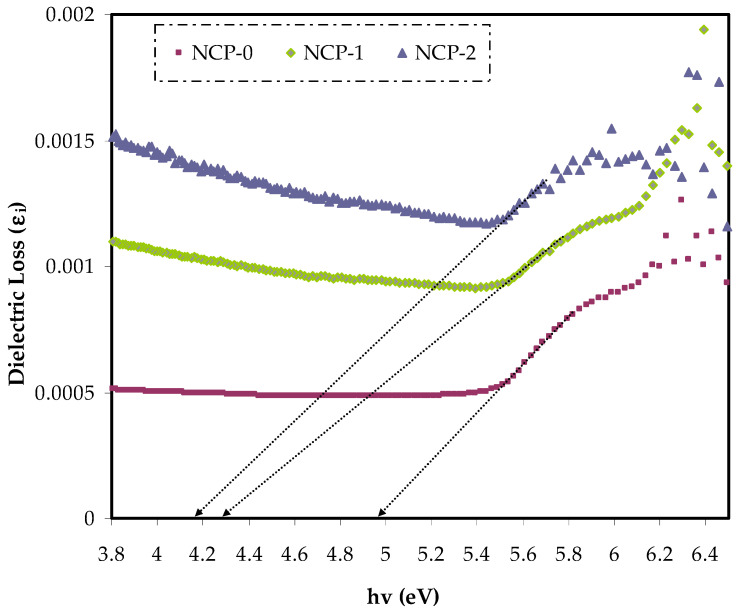
Dielectric loss spectra v wavelength for NCP-0 and composite films.

Based on the quantum view, the optical dielectric loss parameter strongly related to the unoccupied and occupied electronic levels in a material. The optical dielectric loss was also largely band gap energy dependent [42]. The optical dielectric loss (*ε_i_*) and photon energy relationship of parent PEO and PEO/CaTiO_3_ samples are shown in Figure 7. From the curve analysis, the optical band gap was obtained from the interception of the extrapolation linear part of the diagram of *ε_i_* that was plotted against the photon energy (*hυ*) with abscissa [43]. It is worth mentioning that Tauc’s formula could estimate the most probable electron-band-gap electron accurately. Table 2 presents the band gap values obtained from the optical dielectric loss plot.

Accurate measurement of the energy band gap provided a clear insight into electron transitions in the band gap structure. Thereby, the optical band gap energy can be considered as a revealing of the optical transition in PEO/CaTiO_3_ nanocomposite film. The band gap energy was obtained from Tauc’s equation for the parent PEO and related nanocomposite films from the α spectra:

(4) $$\left(\alpha hv\right)=B{\left(hv-E_{g}\right)}^{\gamma }$$

**Figure 8:**
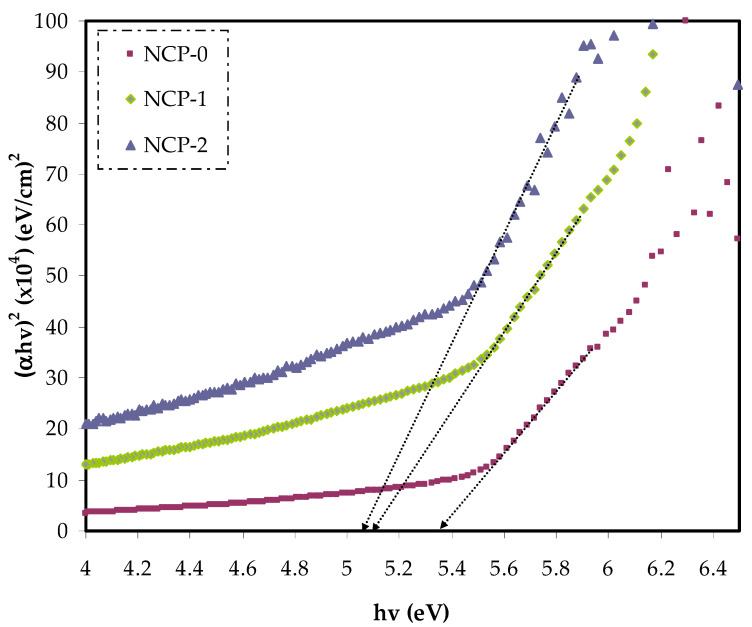
(*αhυ*)^2^ v photon energy for NCP-0 and composite samples.

**Figure 9:**
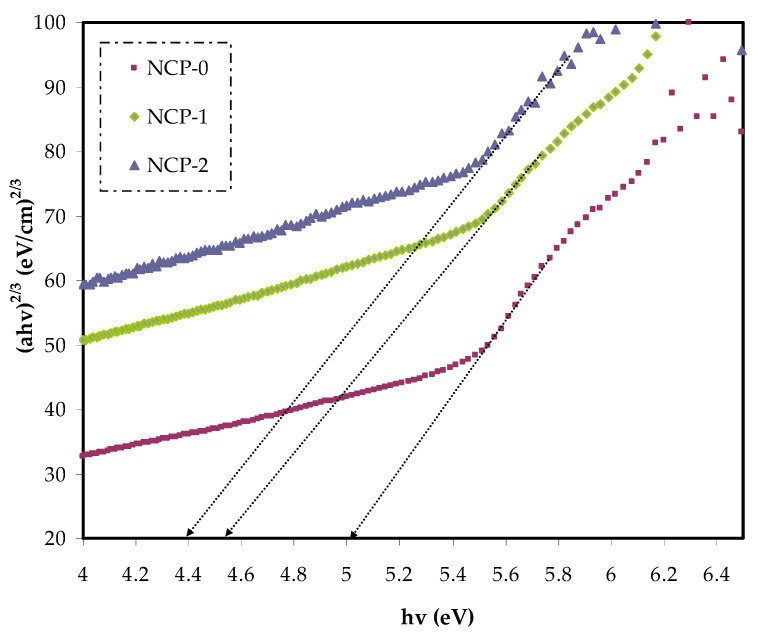
(*αhυ*)^2/3^ v photon energy for NCP-0 and composite samples.

**Figure 10:**
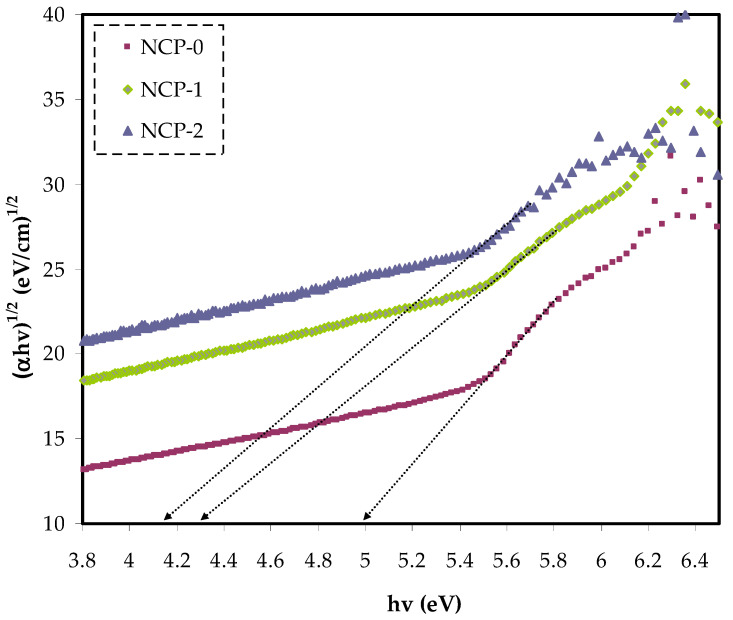
(*αhυ*)^1/2^ v photon energy for NCP-0 and composite samples.

**Figure 11:**
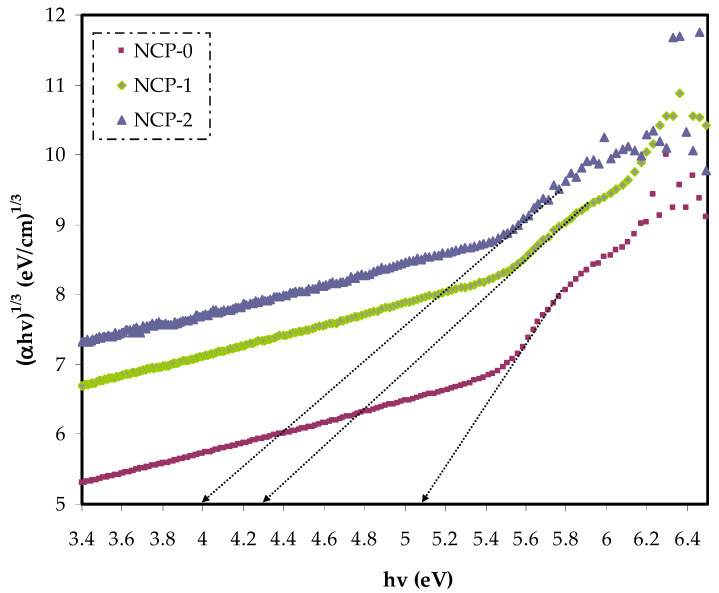
(*αhυ*)^1/3^ v photon energy for NCP-0 and composite samples.

In Equation (4), the parameter reliant on the interband transition probability is *B*. The incident energy is expressed as *hυ*. *Eg* symbolizes the energy band gap, and the index γ defines the kind of electron transition [44]. The value of direct allowed electron transitions (γ) was ½, the γ for indirect allowed transition was 2, for direct forbidden transitions γ was 3/2, and for the indirect forbidden transitions γ was 3 [45]. For determination of *E_g_* value, for the electronic transitions of the direct allowed (Figure 8), direct forbidden (Figure 9), indirect allowed (Figure 10), and indirect forbidden (Figure 11), the intersect of the extrapolated linear part of the diagram of (*αhυ*)^1/γ^ v *hυ* with abscissa was used [46]. In earlier research, it was suggested that the cause of decreasing the optical energy band gap could be due to the diverse localized trap states through forbidden band gap. These localized levels were created from the loaded nanoparticles to the polymer [47,48].

The creation of new energy states within the band gap can be developed by introducing defects (deep- and tail-localized levels). Thus, the migration of electron transition from the valence to conduction band, supported lowering band gap energy [48]. From both the cut-off energy extracted from the dielectric loss and band gap energy, we recognized the greatest possible electronic transition in each sample [49]. Based on the energy band gap values extracted from Tauc’s equation (Figure 8, Figure 9, Figure 10 and Figure 11) and cut-off energy extracted from the dielectric loss plot, the direct transition (γ = 1/2) was the most probable electron transition (see Table 2). Summarily, from comparing the value of the optical band gap derived from Tauc’s plot and that calculated from the dielectric loss diagram, the maximum probable electron transitions and the optical band gap value could be determined.

### 3.5. Optical Dielectric Properties

**Figure 12:**
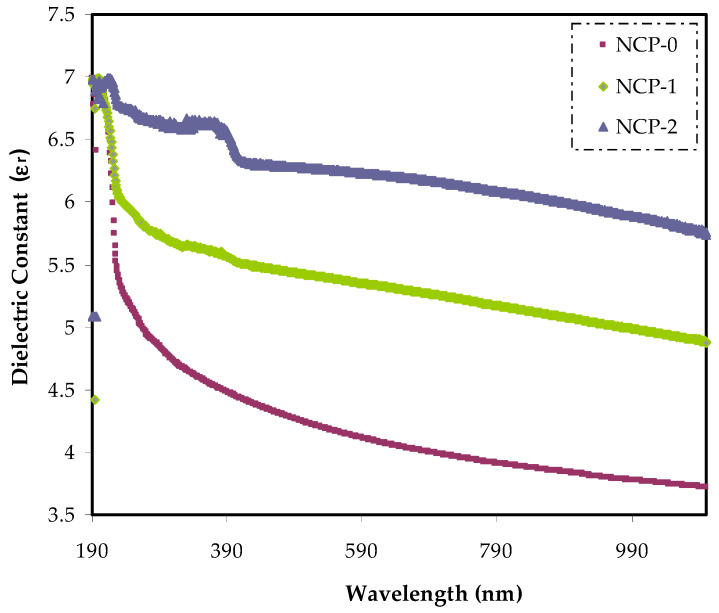
Dielectric constant vs. wavelength for NCP-0 and composite films.

The basic meaning of dielectric characteristic is a reflection of the material’s optical property [41]. The dependence of the dielectric constant on energy indicated that definite electron–photon interaction within the energy range in the film were generated. These new interactions determined the responses in the imaginary and real parts of the dielectric spectra, appearing as peaks [50]. It was concluded that the imaginary and real parts were not only in association with the refractive index, but also dependent on the extinction coefficient by Equation (5) [51]:

(5) $$\varepsilon_{r}=n^{2}-k^{2}=\varepsilon_{\infty }-\frac{e^{2}}{4\pi C^{2}\varepsilon_{o}}\frac{N}{m^{*}}\lambda^{2}$$

where, the dielectric constant at relatively high wavelengths and the free-space dielectric constant are denoted by *ε*_∞_ and *ε_o_*, respectively. The ratio between localized density state and effective mass is referred to by *N*/m^*^. The electronic charge is symbolized by *e*. The relaxation time is represented by *τ*, and the light velocity is referred to by C. The dielectric constant (*ε’*) spectra are displayed in Figure 12. The optical dielectric constant related to the wavelength for every sample, and it was clearly seen that increasing the CaTiO_3_ filler ratio caused an elevation in the *ε’* value from 3.7 to 6.29. More profoundly, the main cause of increasing *ε’* value was the introduction of energy states. In other words, there was a direct connection between ε’ and states density inside the forbidden gap of polymer samples [31,32].

Using *ε_i_* extrapolation at the plateau region to the *Y* axis was useful in extracting the *ε_i_* of the PEO and 4 and 8 wt.% of PEO/CaTiO_3_ nanocomposite samples. Incorporating the CaTiO_3_ nanoparticles into PEO increased the *ε_i_* value as a consequence of creating new states. In other words, there was an existence between the *ε_i_* and density of states through forbidden gaps in the materials [52,53].

Optical properties can be achieved by studying conduction, dispersion, reflection, absorption, and polarization phenomena. In optoelectronics and solar cell devices, the use of direct band gap semiconductor perovskite materials is essential. The direct band gap semiconductors revealed the extent of absorbing energy (photon) by electrons moving directly to the conduction band. In the case of indirect band gap semiconductor materials, a phonon created heat influence and lowered the reliability of the devices. Furthermore, the interband transition were better than the intraband transition in the large band gap semiconductor. In former ones, there are transitions from the valence band to the conduction band, deep state, and shallow state transition [54].

### 3.6. Urbach Energy as a Measure of Order or Disorder

**Figure 13:**
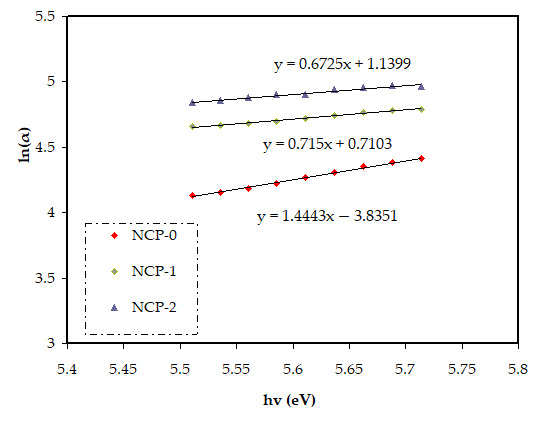
Urbach plot for NCP-0 and composite films.

Figure 13 displays the Urbach diagram for the PEO and PEO-loaded samples. From the plot, the prediction of whether the samples were crystalline or amorphous after doping was decided. The Urbach tail was seen in amorphous and disordered materials and was vital for realizing the electronic passage property of these materials. It can be proved that the band tail levels in the amorphous structure resulted from strains in the network that were adequate to thrust the levels to the forbidden band gap. Interestingly, the tails decayed exponentially into the band gap [52,55]. At relatively low absorption levels, the absorption coefficient (*α*) was best described by the Urbach formula [56]:

α = α_o_
*exp*(−hω/ΔE) (6)

where *α_o_* refers to a constant and ΔE denotes energy, which was obtained from the tail width of localized levels inside the forbidden gap. The resultant of state tailing at the Urbach band edges came from the disorder of the structure. These tail states indicated the contribution of the photon energy absorption below the energy band gab, i.e., the tail states were characteristic behavior of absorption in the sub-gap region [56]. As Saq’an et al. stated, the Urbach energy decrement was sufficient evidence of growing the crystalline structure. Prasher et al. emphasized that when Urbach energy increases, it indirectly indicates a growth in the amorphous portion [55]. All of this demonstrated the accuracy of Urbach energy in predicting the structure of solid materials, i.e., the structure of samples transferred from crystalline to amorphous beyond the doping. The Urbach energy of parent PEO and loaded PEO are listed in Table 3. The Urbach energy increased from 0.692 eV to 1.487 eV parent PEO and the PEO doped with 8 wt.% of CaTiO_3_ particles, respectively. This shift in energy revealed the growth of the amorphous region. In the present study, a lowering in intensity and increase in the broadness of the diffraction peak of pure PEO were recorded after adding CaTiO_3_ particles.

### 3.7. Optical Microscopy Study of PEO Morphology

**Figure 14:**
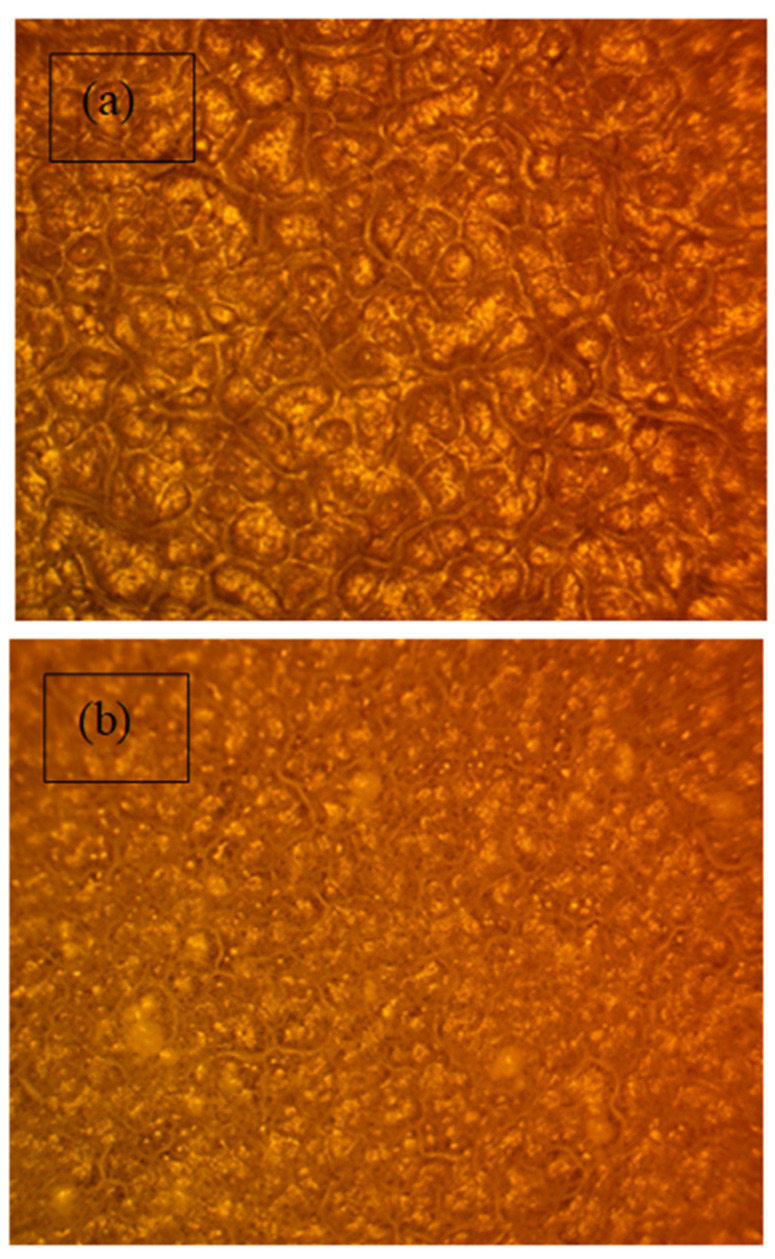

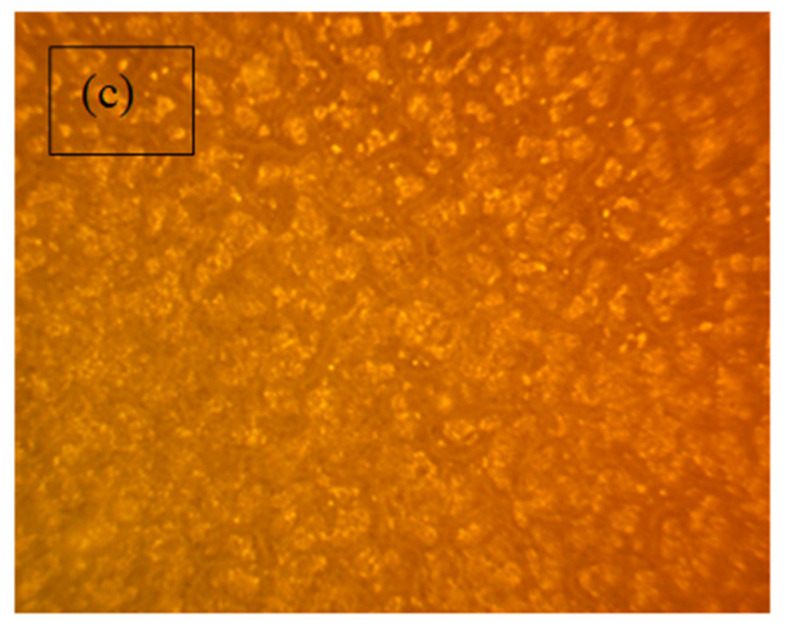
Optical microscopy images for (**a**) Pure PEO, (**b**) NCP-1, and (**c**) NCP-2 composite films.

PEO-based polymer with high molecular weight and polycrystalline materials with micron-sized randomly aligned distinct domains (See Figure 14a). Each domain was made up of aligned or tangled chains characterized by a wide range of topological interactions. Physical crossings, knotting, and looping were examples of such interactions [57,58]. It is worth noting that pure PEO electrolytes contain semi-crystalline and amorphous regions and intermediate regions at the crystalline/amorphous interphase, below the melting temperature (≈ 330 K). At ambient temperature, PEO’s semi-crystalline structure resulted in both amorphous and crystalline phases [38,59,60,61]. In pure PEO samples, some spherulites of varying sizes were generated by lamellar eutectics, as seen in Figure 14a. Spherulites covered a larger surface area in pristine PEO than what was found in doped samples. Because spherulites were the crystalline phase of PEO, this was evidence of increased crystallinity relative to doped samples [62,63]. The composite film became smoother as the fraction of CaTiO_3_ increased, spheroids’ size decreased, and individual spheroids’ boundaries became apparent (see Figure 14b,c). The literature suggests that, spheroids form through random nucleation and develop radially until they collide at borders, as shown in Figure 14a. The morphological behavior of crystalline polymers with flexible chains was characteristic. If we closely look at one of the spheroids, one can see that it has more fiber texture (amorphous phase) than the others [64].

## 4. Conclusions

In conclusion, the construction of direct band gap polymer composites with improved amorphous phase showed the efficiency and eligibility of the polymer for application in optoelectronic devices. Implementation of the solution-cast technique was carried out in the construction of PEO/CaTiO_3_ nanocomposites. Development of amorphous nature in parent PEO with CaTiO_3_ was evidenced via XRD. The refractive index dispersion behavior of PEO was shifted to a greater wavelength by loading with CaTiO_3_. The optical properties of the PEO-based composites had a small optical energy band gap close. Both the refractive index and dielectric constant were significantly modified when an optimum quantity of CaTiO_3_ was added into the PEO polymer. Taucs model was successfully applied in determining the type of electronic transition within the samples. The desired optical energy band gap was achieved from the analysis of the dielectric loss parameter. The improvement in band gap and flexibility of films that were achieved, were potentially usable in optoelectronic devices. Summarily, the most significant possible electron transition type was determined from the diagram of dielectric loss. This development of UV-Vis absorption also made the prepared nanocomposite film the candidate of choice in future nanotechnology-based device utilization.

## Figures and Tables

**Table 1:** Absorption edge for NCP-0 and composite films.

Samples	Absorption Edge (eV)
NCP-0	5.2
NCP-1	4.65
NCP-2	4.4

**Table 2:** The *E_g_* values from Tauc’s method and *ε_i_* plot.

Films	γ = 3/2	γ = 2	γ = 1/2	γ = 3	Dielectric Loss
NCP-0	5.1	5	5.38	5.08	4.90
NCP-1	4.58	4.3	5.15	4.3	4.28
NCP-2	4.4	4.18	5.13	4	4.19

**Table 3:** Urbach energy for pure PEO and PEO doped solid polymer films.

Sample Designation	Urbach Energy (eV)
NCP-0	0.692
NCP-1	1.398
NCP-2	1.487

## Data Availability

The data presented in this study are available on request from the corresponding author.
